# Supplementary material for: The interactome of intact mitochondria by cross-linking mass spectrometry provides evidence for coexisting respiratory supercomplexes
Source: Mol Cell Proteomics. 2017 Dec 8;17(2):216–32. doi: 10.1074/mcp.RA117.000470 (PMC5795388; doi:10.1074/mcp.RA117.000470)
Supplement: Supplemental Data [file supp_RA117.000470_133922_0_supp_23978_fzffwf.zip › spectra_annotation/mito_spectra_annotation/487-1-4-1-12-1.pdf]

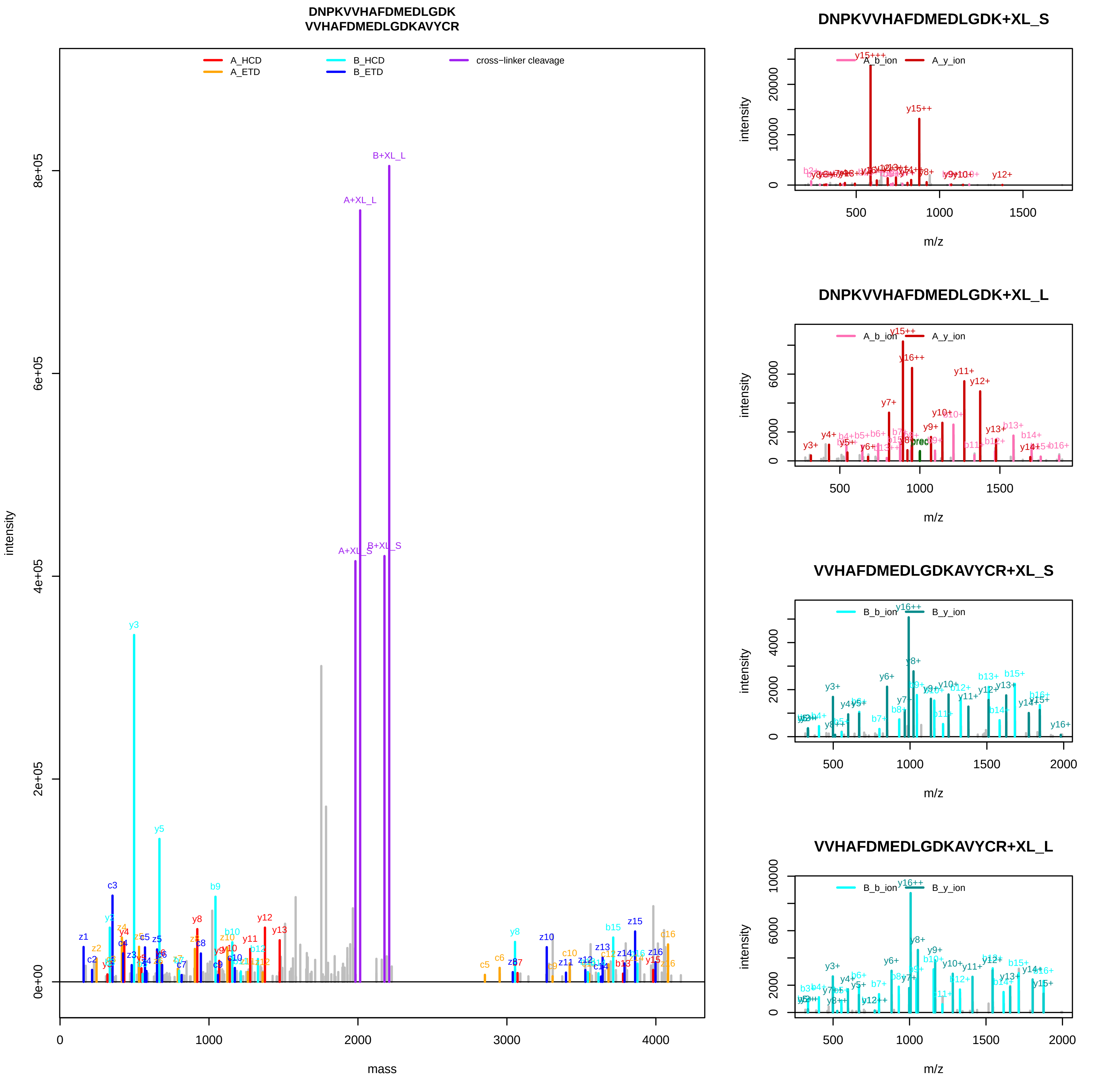
DNPKVVHAFDMEDLGDK
VVHAFDMEDLGDKAVYCR
A_HCD
A_ETD
B_HCD
B_ETD
cross−linker cleavage
B+XL_L
A+XL_L
A+XL_S
B+XL_S
y3
y5
c3
b9
y8
y12
y13
b15
z15
c16
intensity
mass
0e+00
2e+05
4e+05
6e+05
8e+05
0
1000
2000
3000
4000
DNPKVVHAFDMEDLGDK+XL_S
A_b_ion
A_y_ion
y15+++
y15++
y12+
DNPKVVHAFDMEDLGDK+XL_L
y15++
y16++
y11+
y12+
y7+
y10+
y3+
y4+
VVHAFDMEDLGDKAVYCR+XL_S
B_b_ion
B_y_ion
y16++
y8+
y6+
y3+
b13+
b15+
y16+
VVHAFDMEDLGDKAVYCR+XL_L
B_b_ion
B_y_ion
y16++
y8+
y9+
y3+
m/z
500
1000
1500
2000
